# Migrated Intravesical Intrauterine Contraceptive Devices: A Case Series and a Suggested Algorithm for Management

**DOI:** 10.7759/cureus.12987

**Published:** 2021-01-29

**Authors:** Omar S Akhtar, Sabahat Rasool, Syed Sajjad Nazir

**Affiliations:** 1 Department of Urology, Government Medical College, Srinagar, Srinagar, IND; 2 Department of Obstetrics and Gynaecology, Government Medical College, Srinagar, Srinagar, IND

**Keywords:** intrauterine contraceptive device, bladder calculus, vesical calculus, migrated intrauterine device, contraception

## Abstract

Introduction

Intrauterine contraceptive devices (IUCD) are a commonly used, reversible, contraceptive method. Complications from insertion rarely include migration into the bladder. We report on two cases of intravesical migrated IUCD and present an algorithm for management based on recently published data.

Materials and Methods

The case records of two patients who underwent surgical procedures for migrated IUCD into the bladder were reviewed. A Pubmed search was performed to identify similar studies. A total of 25 papers met the criteria for inclusion.

Results

Both cases were managed with laparotomy and partial cystectomy. A review of literature suggests recently reported cases of IUCD migration are rising, with most cases having been reported in the last decade. Bladder calculus developing over the migrated IUCD is the most common presentation. Most cases have been managed using endourological techniques. A small number of cases have required open vesicolithotomy or laparoscopic surgery. Rarely, laparotomy has been required.

Discussion

IUCD migration into the bladder remains rare, however, recently the number of reported cases has risen. A thorough physical examination and radiological evaluation are warranted. Management is surgical in all cases. Most cases can be managed with endourological techniques. A treatment algorithm has been suggested in this paper based on recent data.

Conclusion

With the rising use of contraception worldwide, the incidence of IUCD migration is possibly going to increase. Treating doctors need to be aware of the possible complications that may arise from a migrated IUCD, including bladder calculi.

## Introduction

Intrauterine contraceptive devices (IUCD) are a popular method of contraception used by approximately 14.3% of women worldwide [1]. Insertion of an IUCD carries a risk of perforation in 1/1000 cases [1]. Migration of IUCD usually occurs after a uterine perforation, which may occur at the time of implanting the device, called primary perforation, or many years later, due to infection or device-related inflammation, called secondary perforation [2]. Migration into the peritoneal space is most reported [3,4]. Migration of the IUCD into the urinary tract is rare and has been reported in only a few dozen cases in the published literature as of 2020. When an IUCD migrates into the bladder, it may cause a local reaction, and deposition of calcium when it enters the lumen. This may progress and form a calculus over many years [5].

In this paper, we examine two cases of spontaneous IUCD migration into the bladder and then review the literature on the management of this rare complication.

## Materials and methods

The case records of two patients who had bladder involvement of migrated IUCD were studied. For a literature review, a search of terms, ‘Intrauterine contraceptive device migration,’ ‘Urinary tract complications of intrauterine contraceptive devices,’ ‘Intrauterine contraceptive device migration in urinary bladder,’ ‘Intravesical intrauterine device migration,’ and ‘Urinary bladder intrauterine contraceptive device,’ were performed in Pubmed. Relevant studies in the English language were perused and studied and data collected.

## Results

Case 1

This is a case of a 40-year-old female patient who was referred with a lost IUCD. On presentation, there was no fever, no urinary symptoms, and no menstrual irregularities. A physical examination was normal. An X-ray revealed the IUCD (Copper-T) to be displaced outside the area of the uterus but within the pelvis. Ultrasonography (USG) performed confirmed that the uterine cavity was empty and showed an echogenic shadow suggestive of an IUCD on the right-side of the uterus. The kidneys, ureters, and bladder were normal. The patient was taken up for laparoscopic removal of the IUCD. During surgery, the IUCD was seen in the right parametrium, densely covered with adhesions. On mobilizing the IUCD, the IUCD was grasped but did not come out. A limb was found embedded in the right, lateral bladder wall. An urgent urological consult was called for. The procedure was converted into a laparotomy and the bladder mobilized on the right side. The IUCD limb was then isolated and the bladder wall marked with electrocautery, which was deepened using sharp dissection until the mucosa. The bladder wall and the IUCD were removed in total and the bladder wall repaired in three layers with absorbable sutures. A Foley catheter along with an intra-peritoneal drain was left in situ. The patient made an uneventful recovery.

Case 2

**Figure 1:**
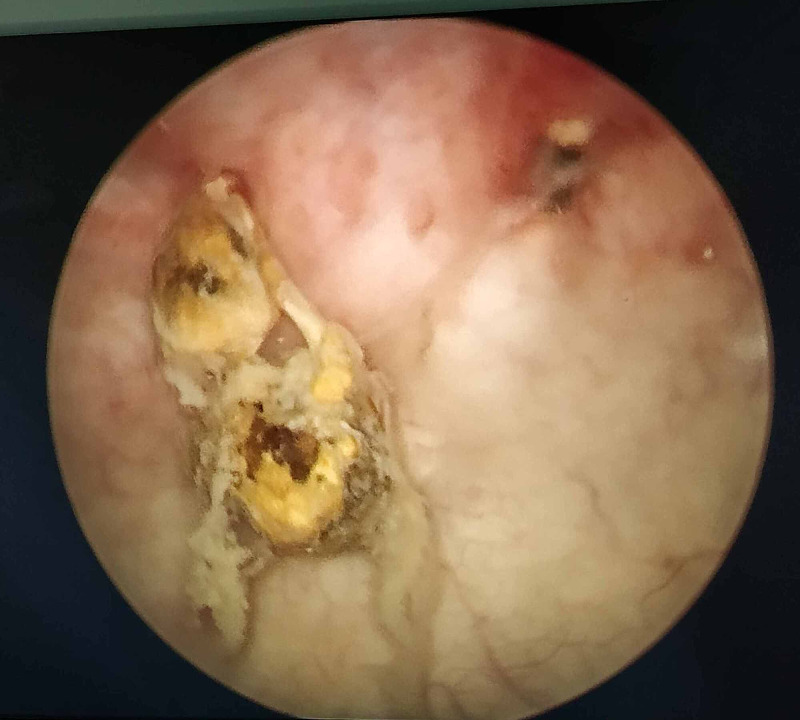
Cystoscopic image of Intrauterine contraceptive devices (IUCD) embedded in the bladder wall.

**Figure 2:**
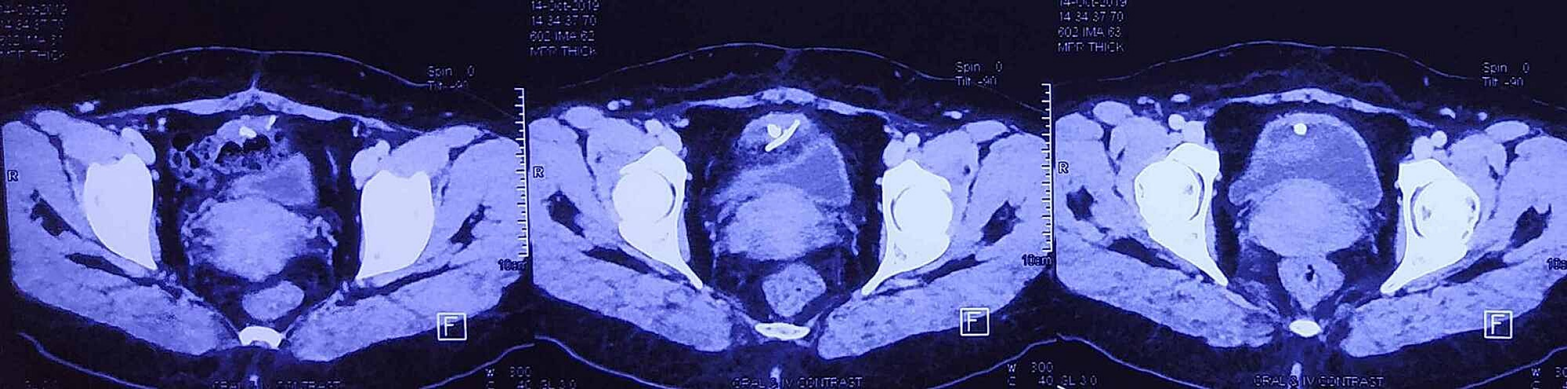
CT scan showing the Intrauterine contraceptive devices (IUCD) within the bladder lumen and traversing the bladder wall

**Figure 3:**
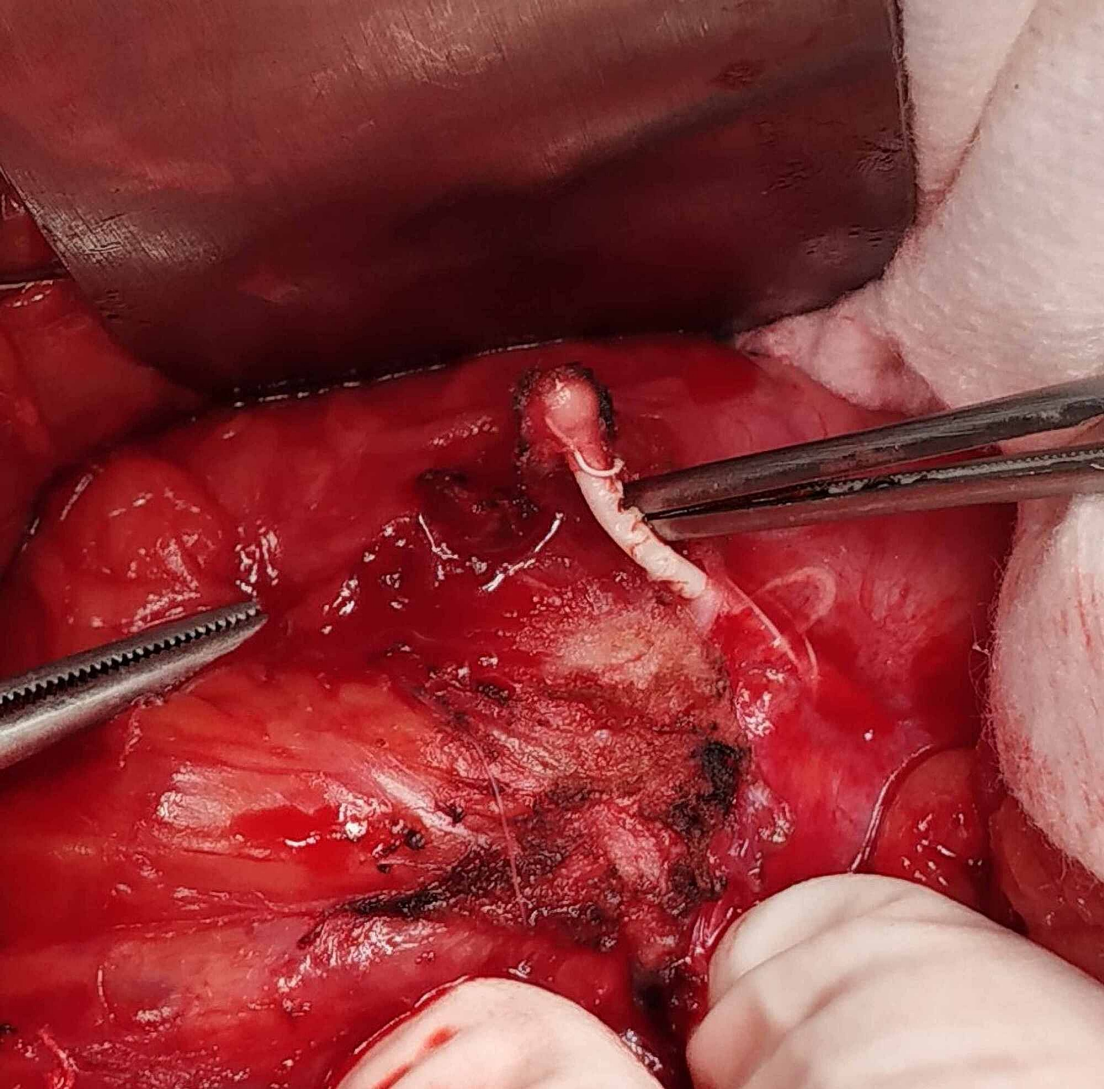
Intra-operative photograph showing the Intrauterine contraceptive devices (IUCD) in the anterior bladder wall

**Figure 4:**
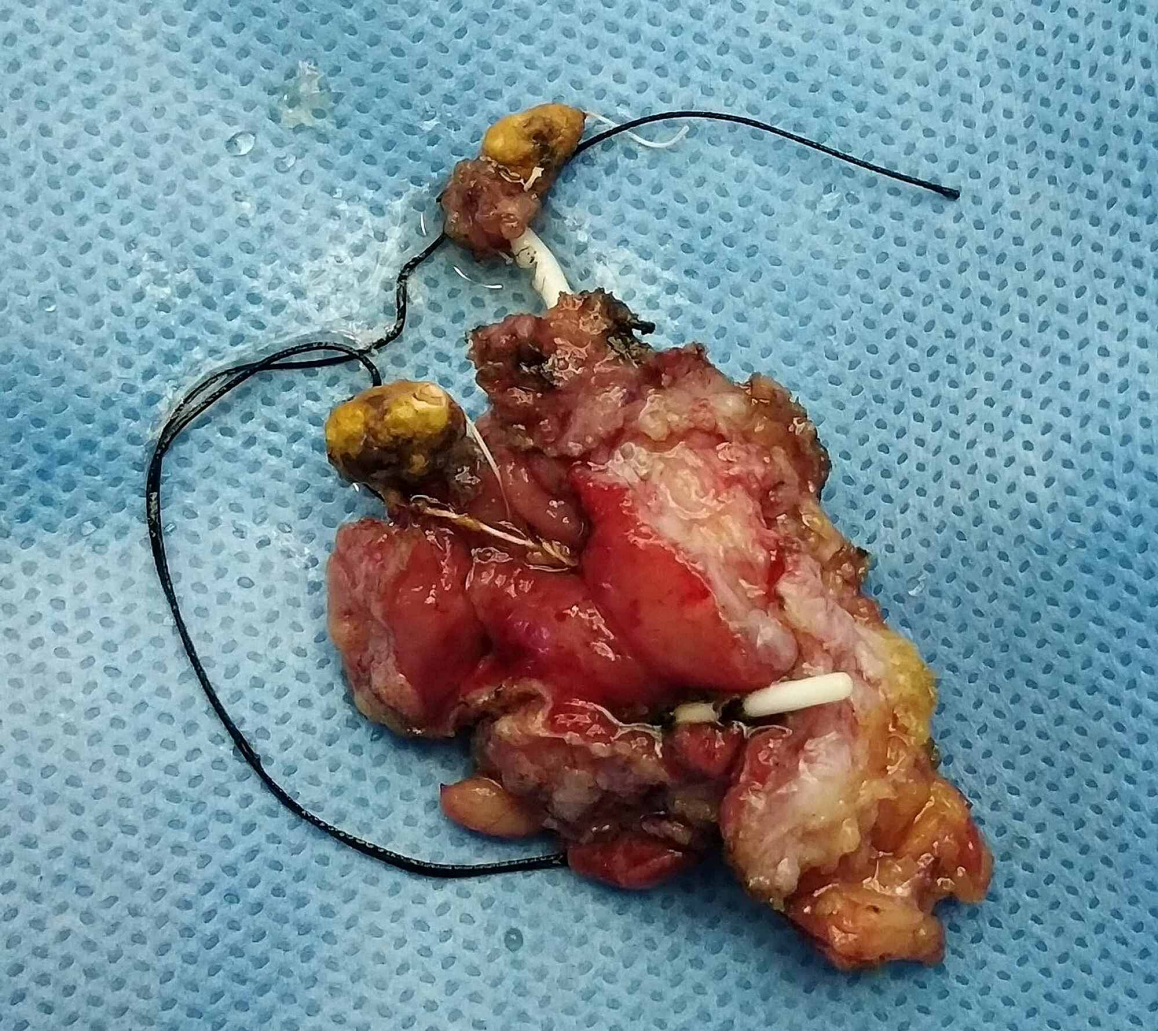
Post-operative photograph of the excised bladder tissue with the Intrauterine contraceptive devices (IUCD) within the bladder wall

A 35-year-old, para 3 patient presented with dysuria, frequency, and urgency. On history, she revealed that she had had an IUCD insertion around a year prior to the presentation but had forgotten about it. At the time of presentation, she was amenorrhoeic for three months, and a pregnancy test was positive. A USG performed showed a bladder calculus of about 1 cm in size. She was taken up for cystoscopic removal of the calculus. However, the calculus was found to be adherent to the bladder wall, and on gentle traction, revealed a limb suggestive of an IUCD limb (Figure 1). The patient was advised surgery, but as there was a risk to the fetus, she elected to postpone the surgery until after the delivery of the child. After completion of term, she was taken up for cesarean section delivery but again elected to postpone the IUCD surgery until after the baby was older. She came for follow-up six months after the cesarean section and was re-investigated at the time. A contrast-enhanced CT scan was performed to rule out any other adjacent organ involvement (Figure 2). An elective exploratory laparotomy was performed, in which intra-operative findings revealed an anteriorly displaced IUCD, which was densely adherent to the anterior bladder wall. (Figure 3). A partial cystectomy was performed with excision of the adjacent bladder wall and the IUCD was removed in toto (Figure 4). A bladder repair was performed. An intraperitoneal drain, a supra-pubic catheter, and a Foley catheter were left in situ. The patient made an uneventful recovery and was symptom-free one year after surgery. 

The details of the patients are noted in Table 1.

**Table 1:** Characteristics of the patients

	Age (years)	Presenting symptom	Complication of IUCD	Management
1	40	Lost threads of IUCD	Migration into bladder wall	Laparotomy + partial cystectomy
2	35	Lower urinary tract symptoms	Bladder calculus on migrated IUCD	Laparotomy + partial cystectomy

Studies which have described IUCD devices that have perforated into the bladder have been mentioned in Table 2. In 1999, Kassab et al reported an extensive literature search and found 23 instances of IUCD perforation into bladder (out of 165 perforations reported at the time). Out of the studies analyzed in the Pubmed search, a total of 25 relevant studies were included, as they had details on patient characteristics, time since insertion to presentation, presentation of patient, and details and outcome of management. In these, patients were identified. The most common presentation was a bladder calculus forming over a migrated IUCD (24/31 patients, 78%). The second most common were embedded IUCD (outside the bladder 3/31 patients, 9.6%), and IUCD in the bladder without a calculus (3/31, 9.6%). Ureteric obstruction was reported in one patient (1/31, 3.2%). The management was surgical in all cases (32/32, 100%). Cystoscopic retrieval (including cystolitholapaxy) was the most common in 16 cases (53%), followed by open vesicolithotomy in seven cases (24%). No major intra-operative or post-operative complications were reported in any of these studies. 

**Table 2:** Review of relevant papers on IUCD migration into bladder

	Authors	Year	Patient(s)	Complication	Time since insertion of IUCD	Management
1	De Silva et al [6]	2017	One	Bladder calculus on migrated IUCD	15 years	Open vesicolithotomy
2	Sano et al [7]	2017	One	Bladder calculus on migrated IUCD	> 2 years	Laser lithotripsy
3	Sharma et al [8]	2017	One	Bladder calculus on migrated IUCD	3 years	Cystoscopic removal
4	Cheung et al [9]	2018	One	Migrated IUCD on bladder surface	3 months	Laparotomy
5	Shin et al [10]	2011	One	Bladder calculus + embedded IUCD	10 years	Laparoscopic excision
6	Waqar et al [1]	2020	One	Bladder calculus on migrated IUCD	10 years	Laser lithotripsy + transvaginal removal
7	Priyadarshani et al [11]	2017	One	Ureteric erosion + obstruction	2 years	Laparotomy + Ureteric reimplantation
8	Tan et al [12]	2019	One	Bladder calculus + embedded IUCD	13 years	Laparotomy
9	Alabi et al [13]	2018	One	Bladder calculus + embedded IUCD	17 years	Laparoscopic + cystoscopic removal
10	Al-Awadi et al [14]	2011	One	Bladder calculus on migrated IUCD	25 years	Open Vesicolithotomy
12	Olaore et al [15]	1999	One	Migration of IUCD into bladder	1 year	Cystoscopic removal
13	Amin and Mehmood [16]	2009	One	Bladder calculus on migrated IUCD	10 years	Open Vesicolithotomy
14	Rafique [17]	2002	One	Bladder calculus on migrated IUCD		Cystoscopic removal
15	Ahmed and Ogunleye [18]	2013	One	Bladder calculus on migrated IUCD	10 years	Open Vesicolithotomy
16	Bashir et al [19]	2016	One	Bladder calculus on migrated IUCD	12 years	Open Vesicolithotomy
17	Jeje et al [20]	2012	One	IUCD migrated into bladder wall	20 years	
18	Ko et al [21]	2011	Two	1. Migrated into bladder wall 2. Migrated into bladder		1. Cystoscopic retrieval 2. Cystoscopic retrieval
19	Aggarwal et al [22]	2014	One	Bladder calculus on migrated IUCD	5 years	Open Vesicolithotomy
20	Nouira et al [5]	2007	Six	Bladder calculi All migrated IUCD’s		Cystoscopic retrieval in all
21	Basiri et al [23]	2019	One	Bladder calculus on migrated IUCD	11 years	Cystoscopic excision from bladder wall
22	Christodoulides [24]	2020	One	Bladder calculus on migrated IUCD	20 years	Cystoscopic removal
23	Ozcelik et al [25]	2003	One	Bladder calculus on migrated IUCD	6 months	Cystoscopic retrieval
24	Dede et al [26]	2006	One	Bladder calculus on migrated IUCD	~ 5 years	Laparoscopic + cystoscopic retrieval
25	Pare et al [27]	2020	One	Migration into bladder	18 months	Cystoscopic retrieval

## Discussion

IUCD is a widely accepted method of contraception. It is easily inserted, is reversible by removal, and causes few side effects [1]. The common side effects are abdominal pain, and heavy menstrual bleeding, especially in the first few months after insertion. Rarely, expulsion, menorrhagia, dysmenorrhoea, pregnancy, and abortion may occur.

IUCD’s can perforate the uterus and then migrate into the pelvic or abdominal spaces. IUCD perforations have been divided into four types according to the anatomical spaces affected. The first compartment is the uterine cavity (type 1), the second is when the IUCD is confined to the myometrium (type 2) and the third compartment is when the peritoneal cavity is breached (type 3). When an IUCD penetrates the surrounding viscera, the perforation is type 4 [6]. 

A uterine perforation may be primary or secondary. A primary perforation occurs at the time of insertion, whereas a secondary perforation occurs after a delay, probably due to pressure necrosis and inflammation of the uterine wall. [2, 28, 29]

IUCD migration may follow uterine perforation. It is a rare complication, occurring between 1.2 - 1.6 per 1,000 insertions [8] Mechanisms that explain migration of an IUCD include iatrogenic perforation, spontaneous uterine contractions, involuntary bladder contraction, gut peristalsis, and peritoneal fluid movement which together contribute to the migration and implantation of the IUCD in other adjacent organs. IUCD’s have most commonly been found in the Pouch of Douglas. They have been found in the ceacum, the bladder, and adjacent to the ureter. Kassab reported 165 perforations of the IUCD with the IUCD located in various organs [3].

IUCD migration into the bladder is a rare complication and most commonly occurs between two and 10 years after implantation. In the first case of this series, the migration was detected three years after insertion, and in the second case, migration was detected after 12 months.

After being in the bladder for a long time, encrustations form over the limbs of the IUCD which can then form a vesical calculus [5]. Rarely, the IUCD can embed in the wall of the bladder and be difficult to remove, necessitating a cystotomy or a partial cystectomy [9].

The initial approach to surgery in the first case was laparoscopic. However, due to dense adhesions between the IUCD and the surrounding tissue including the bladder, conversion to laparotomy was required. A partial cystectomy was needed in this patient. Shin et al demonstrated the use of laparoscopic approach alone to manage an embedded IUCD [10]

Sharma et al performed a cystoscopic retrieval of an intravesical IUCD [11]. Sano et al have described a case in which laser lithotripsy was used to remove a bladder calculus under general anaesthesia [12]. In the second case of our series, this was attempted, but the limbs of the IUCD were embedded in the wall of the bladder and covered with a calculus and the procedure could not be safely performed. This necessitated a thorough evaluation and subsequent laparotomy.

Of the twenty-six papers that have been cited in Table 2, 18 papers (69.2%) have been published in the last decade alone. A growing world population along with an increase in the use of contraception worldwide, as is evidenced by falling birth rates, translates to a potential increase in the incidence of IUCD migration in the coming years. Doctors treating women with potential complications of IUCD insertion need to be aware of this fact.

**Figure 5:**
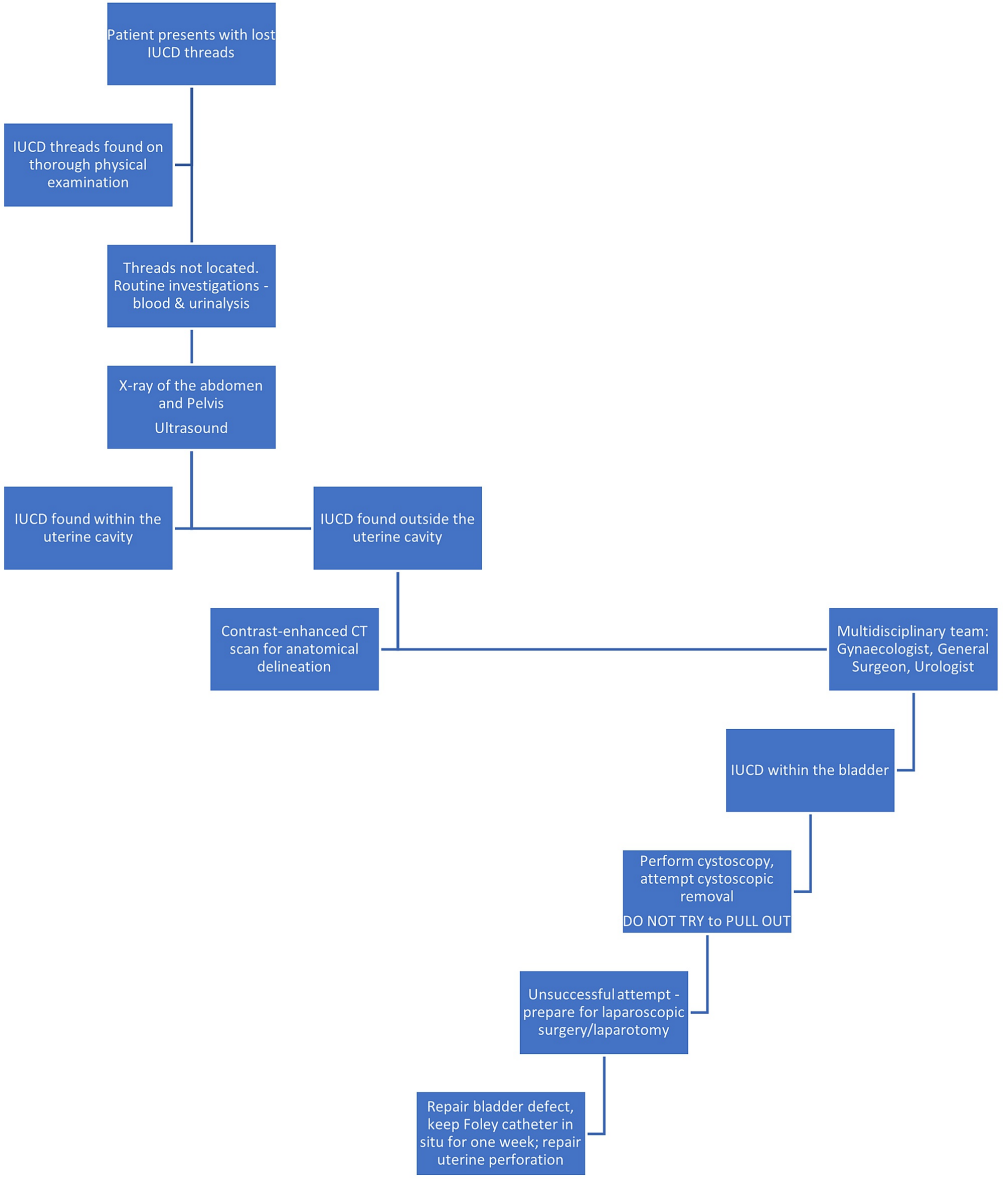
A suggested algorithm for management of migrated IUCD

Based on the published data, an algorithm is suggested for the management of patients with migrated IUCD’s that may involve the urinary tract (Figure 5).

## Conclusions

Most IUCD migrations occur at the time of insertion, and proper training of healthcare workers is imperative to prevent complications. Although rare, IUCD migration is a complication with high morbidity. IUCD migration into the bladder is a debilitating condition for the patient and warrants a multi-disciplinary approach with the use of imaging techniques and cystoscopy to locate the IUCD. Proper patient preparation is vital to a successful outcome. With an increasing number of women worldwide adopting some form of contraception, including IUCD, the incidence of migrated IUCD’s is going to rise in the future, and gynecologists, surgeons, and urologists need to be aware of this complication.
